# Multi-genome comparative analysis and VIGS validation suggest that *GhMAPKKK5* negatively regulates fruiting branch angle in upland cotton

**DOI:** 10.3389/fpls.2026.1929470

**Published:** 2026-09-16

**Authors:** Wenjiao Zhang, Wenmin Yuan, Panfeng Yao, Yarong Zhou, Junji Su, Qi Ma, Caixiang Wang

**Affiliations:** 1 State Key Laboratory of Aridland Crop Science, College of Life Science and Technology, Gansu Agricultural University, Lanzhou, China; 2 Cotton Research Institute, Xinjiang Academy of Agricultural and Reclamation Science/Key Laboratory of Cotton Biology, Genetics and Breeding in Inland Northwest China, Ministry of Agriculture and Rural Affairs/Xinjiang Production and Construction Corps Key Laboratory of Crop Germplasm Enhancement and Biobreeding, Shihezi, Xinjiang, China

**Keywords:** fruiting branch angle, *GhMAPKKK5*, multi-genome comparative analysis, upland cotton, VIGS

## Abstract

Plant architecture strongly influences planting density, canopy light interception, and mechanical harvesting efficiency in upland cotton (*Gossypium hirsutum* L.). Fruiting branch angle (FBA) is an important architectural trait, but its molecular regulation remains poorly understood. Here, we compared the MAPK/ERK kinase kinase (MEKK) subfamily across four upland cotton reference genomes and identified 44 MEKK members in each genome. Genomic positions and coding sequences were broadly conserved, whereas motif analysis distinguished 26 motif-conserved from 18 motif-variable homologous groups. Integrating this classification with phylogenetic relationships and expression differences between accessions with contrasting FBA prioritized *GhMAPKKK5* (*GhChrD12G0779*) for functional analysis. *GhMAPKKK5* expression was lower in large-angle accessions than in compact accessions. Virus-induced gene silencing of *GhMAPKKK5* increased FBA at both the squaring-to-flowering and boll-setting stages, providing preliminary evidence that *GhMAPKKK5* negatively influences branch orientation. Silencing was also associated with increased brassinosteroid content, reduced cytokinin-equivalent content, and altered expression of hormone- and branch angle-related genes, including *GhLAZY1*, *GhTAC1*, and *GhSAUR*. Together, these results link *GhMAPKKK5* activity to FBA and to brassinosteroid- and cytokinin-related processes. *GhMAPKKK5* is therefore a candidate for further mechanistic and agronomic evaluation of cotton branch architecture.

## 1 Introduction

Cotton (*Gossypium* spp.) is the world’s most important natural fiber crop and a major source of raw material for the textile industry (Chen et al., 2007). As large-scale, mechanized production expands, particularly in northwestern China, plant architecture has become a central breeding target. Optimized architecture can improve planting density, canopy light interception, defoliation efficiency, and suitability for mechanical harvesting (Li et al., 2020; Ma et al., 2025). Fruiting branch angle (FBA) is a major component of cotton architecture that affects canopy structure, photosynthetic efficiency, dry-matter accumulation, and yield formation (Jiang et al., 2013; Qi et al., 2016; Zheng et al., 2020; Hu et al., 2021; Ye et al., 2024). Defining the genetic and molecular basis of FBA is therefore important for breeding compact cultivars suited to dense planting and mechanical harvesting.

Branch angle formation is regulated by complex networks involving gravitropic signaling, light perception, cell expansion, and phytohormone pathways. In several plant species, *LAZY* and *TAC1* family genes have been shown to regulate branch or tiller angle by modulating gravity responses and asymmetric auxin distribution (Ku et al., 2011; Dardick et al., 2013; Yoshihara and Spalding, 2017; Huang et al., 2021; Xia et al., 2021; Li et al., 2022). Brassinosteroids (BRs) also play central roles in organ angle and plant architecture regulation, as demonstrated by BR biosynthesis and signaling mutants in rice, maize, and wheat (Yamamuro et al., 2000; Bai et al., 2007; Hong et al., 2002; Li et al., 2009; Sakamoto et al., 2006; Tanabe et al., 2005; Tian et al., 2019; Chen et al., 2026). In cotton, genome-wide association studies and transcriptome analyses have identified multiple loci and candidate genes associated with FBA (Xu et al., 2019; Shao et al., 2022; Tan et al., 2025). More recently, *GhFBA1_At* was cloned as a major gene controlling cotton FBA, and the GATA5-GhFBA1_At-KNAT7 module was shown to regulate branch angle by affecting cell expansion and cell wall properties (Xu et al., 2026). Nevertheless, the molecular network underlying cotton FBA remains incomplete, and additional signaling components involved in FBA regulation remain to be identified.

Mitogen-activated protein kinase (MAPK) cascades are conserved signaling modules that regulate plant development, hormone responses, and environmental adaptation. MEKK-type MAPKKKs act upstream in these cascades and have been implicated in plant architecture and hormone signaling (Wu et al., 2014; Zhang and Zhang, 2022). *Arabidopsis YODA*/*MAPKKK4* regulates inflorescence architecture through the *MKK4*/*MKK5*-*MPK3*/*MPK6* cascade (Meng et al., 2012). In rice, *OsMAPKKK10* and *OsMAPKKK5* contribute to panicle development, brassinosteroid signaling, tillering, plant height, and yield-related traits (Guo et al., 2018; Xu et al., 2018; Yan et al., 2025). Although cotton MAPK cascade genes have been catalogued (Yin et al., 2021), their structural conservation across upland cotton genomes and their value for prioritizing architecture-related candidates remain unclear. We therefore compared MEKK genes across four independently assembled upland cotton genomes and classified homologous groups according to motif conservation. We then integrated cross-genome conservation, phylogeny, and expression differences between accessions with contrasting FBA. This approach prioritized *GhMAPKKK5* for expression analysis, VIGS-mediated silencing, hormone profiling, and qRT-PCR analysis of branch angle-related genes. The study provides preliminary functional evidence linking *GhMAPKKK5* to FBA and identifies a candidate for further evaluation of compact cotton architecture.

## 2 Materials and methods

### 2.1 Plant materials and growth conditions

Ten upland cotton (*Gossypium hirsutum* L.) accessions exhibiting contrasting fruiting branch angle (FBA) phenotypes were selected from the Cotton Molecular Breeding Laboratory of Gansu Agricultural University. Five accessions with relatively large FBA, including CCRI23, Jiangsumian1, BJA592, 70-24, and Shaan689, were used as the large-angle group, whereas Xinluzao21, Jimian8, Xinluzao15, Xinluzhong14, and Longmian3 were used as the small-angle group.

Seeds were sown in a 1:1 (v/v) mixture of nutrient soil and vermiculite for germination. The seedlings were cultivated in a controlled growth chamber with a 16 h light/8 h dark cycle, a temperature of 25 °C, and a light intensity of approximately 8,000 lx. Once the cotyledons had fully expanded, the seedlings were transferred to 18 cm × 20 cm plastic pots and grown at a planting density of 20 plants m^-^². All experimental plants were maintained under the same controlled conditions to minimize environmental variation during phenotypic evaluation.

### 2.2 Identification of MEKK subfamily members across four upland cotton genomes

T2T genome assemblies are characterized by high contiguity and completeness, providing high-quality reference sequences for gene family identification and cross-genome comparison (Aoyagi Blue et al., 2025). To establish a multi-genome comparative framework for the cotton MEKK subfamily, four upland cotton reference genomes, including TM-1, Zhongmian113, Jin668, and Nongdamian8, were analyzed. Genome assemblies and annotation files for TM-1 and Jin668 were downloaded from T2TCottonHub, Zhongmian113 genome data were obtained from the Zhongmian113 Genome Database, and Nongdamian8 genomic resources were retrieved from CottonMD.

Following the homology-based identification strategy used in the previous genome-wide characterization of cotton MAPK cascade genes (Yin et al., 2021), the 44 previously reported GhMEKK protein sequences were used as queries to identify the corresponding MEKK homologs in each of the four genomes. Local BLASTP searches were performed using TBtools v2.103 with an E-value threshold of 1 × 10^-5^. Redundant sequences were removed manually, and candidate proteins were further verified by conserved-domain analysis using InterProScan and the NCBI Conserved Domain Database (CDD). Only proteins containing the characteristic serine/threonine protein kinase catalytic domain (S_TKc) were retained for subsequent comparative and evolutionary analyses.

### 2.3 Cross-genome synteny and selective-pressure analyses of MEKK genes

To evaluate the genomic conservation of MEKK loci across the four upland cotton genomes, genome annotation files, protein sequences, and coding sequences (CDS) from TM-1, Jin668, Nongdamian8, and Zhongmian113 were used for cross-genome synteny analysis. Pairwise whole-genome collinearity was analyzed using MCScanX implemented in TBtools v2.103 (Wang et al., 2012; Chen et al., 2020). Whole-genome syntenic relationships were used as the genomic background, and collinear relationships involving the identified *MEKK* genes were subsequently extracted and highlighted. To reduce excessive overlap and improve visualization, MEKK-specific syntenic links were displayed for three sequential genome comparisons: TM-1 versus Jin668, Jin668 versus Nongdamian8, and Nongdamian8 versus Zhongmian113. Whole-genome syntenic relationships were displayed in gray, whereas MEKK-specific links were highlighted using different colors.

To further assess the evolutionary constraints acting on *MEKK* coding sequences, homologous *MEKK* gene pairs were compiled for all six pairwise comparisons among the four genomes. The corresponding coding sequences (CDS) were used to calculate the nonsynonymous substitution rate (Ka), synonymous substitution rate (Ks), and Ka/Ks ratio using the Ka/Ks calculation function in TBtools (Chen et al., 2020). Ka/Ks ratios below 1 were interpreted as evidence of purifying selection, values close to 1 as approximately neutral evolution, and values above 1 as potential indications of positive selection or accelerated sequence evolution (Hurst, 2002). Because Ka/Ks estimates can become unstable when Ks values are very low, elevated Ka/Ks ratios associated with low Ks values were interpreted cautiously. The distributions of Ka, Ks, and Ka/Ks values were visualized as boxplots with individual gene-pair values using the ggplot2 package in R 4.5.1 (Wickham, 2016).

### 2.4 Gene structure and conserved motif analyses

To investigate variation in gene structure among corresponding MEKK homologs, CDS, genomic sequences, and protein sequences of the identified MEKK members were extracted from the corresponding genome annotation files. Gene structures were analyzed using the Gene Structure Display Server (GSDS 2.0), and exon-intron organization was visualized based on the alignment between CDS and genomic sequences.

Conserved protein motifs were identified using MEME Suite v5.5.7, with the maximum number of motifs set to 10 and motif widths ranging from 6 to 50 amino acids, allowing zero or one occurrence of each motif per sequence (Bailey et al., 2009). Gene structures and motif distributions were subsequently integrated and visualized using the Gene Structure View tool in TBtools (Chen et al., 2020). Based on the cross-genome comparison of motif composition and arrangement among corresponding homologs, the 44 MEKK homologous groups were further classified into motif-conserved and motif-variable homologous groups. Homologous groups showing consistent motif composition and arrangement across the four genomes were classified as motif-conserved, whereas those showing loss or positional variation of one or more motifs in at least one genome were classified as motif-variable.

### 2.5 Phylogenetic relationships and promoter *cis*-element analyses

To further characterize the evolutionary relationships among the identified MEKK members, phylogenetic analysis was performed using MEKK protein sequences from the four upland cotton genomes. A Neighbor-Joining (NJ) tree was constructed in MEGA11, and branch support was evaluated using 1,000 bootstrap replicates (Tamura et al., 2021). The resulting phylogenetic tree was visualized and annotated using the Interactive Tree of Life (iTOL v6) platform (Letunic and Bork, 2024).

To explore the potential transcriptional regulatory features of the motif-conserved MEKK members, 2-kb sequences upstream of the translation start site of the corresponding TM-1 homologs were retrieved as putative promoter regions. *Cis*-acting regulatory elements were identified using PlantCARE (Lescot et al., 2002). The detected elements were grouped into four major functional categories: light-responsive, phytohormone-responsive, stress-responsive, and growth/development-related elements. Their composition and distribution were subsequently visualized using TBtools.

### 2.6 Expression analysis and candidate gene screening

Transcriptome expression data from cotton accessions with contrasting fruiting branch angle phenotypes were used for preliminary screening of genes corresponding to the 26 motif-conserved *MEKK* homologous groups. Genes showing relatively large expression differences between the two accessions were selected for further validation.

The selected genes were subsequently examined by qRT-PCR in five relatively large-FBA accessions and five relatively small-FBA accessions. The FBA values of these accessions are provided in **Supplementary Table 2**. Three independent biological replicates were analyzed for each accession, with three technical replicates for each biological replicate. Candidate genes for subsequent functional validation were prioritized based on the combined results of transcriptome screening and qRT-PCR validation.

### 2.7 Construction of VIGS vectors and virus-induced gene silencing

Total RNA from Jimian8 plants was isolated using the FastPure Plant Total RNA Isolation Kit (Vazyme, China). First-strand cDNA was then prepared with HiScript III Reverse Transcriptase (Vazyme). A 511-bp fragment specific to *GhMAPKKK5* was amplified and inserted into the CLCrV vector using restriction enzyme digestion and ligation. Recombinant plasmids were introduced into Agrobacterium tumefaciens strain GV3101. For VIGS assays, cultures containing CLCrV:*GhMAPKKK5*, CLCrV:00, and CLCrVA:*GhCHLI* were mixed with helper vector CLCrVB at a 1:1 ratio and adjusted to OD_600_ ≈ 1.8. Cotyledons of young cotton seedlings were infiltrated using a needleless syringe. Plants exhibiting the characteristic photobleaching phenotype of the positive control CLCrVA:*GhCHLI* were considered successfully infected. Silencing efficiency was evaluated by qRT-PCR. For this analysis, equal amounts of leaf tissue from two individual plants were pooled to form one sample, and each pooled sample was analyzed using three technical qRT-PCR replicates. Plant pairs represented by pooled samples showing *GhMAPKKK5* expression levels reduced to ≤50% of those in the CLCrV:00 control were selected for subsequent phenotypic analyses.

FBA measurements were conducted at both the squaring-to-flowering stage and boll-setting stage. For each plant, lower fruiting branches (1st-3rd branches from the base) and upper fruiting branches (4th-6th branches from the base) were evaluated. FBA was defined as the angle between the fruiting branch and the main stem. Measurements were obtained using a digital angle ruler. Branch length was measured from the branch base to the terminal reproductive node. At least ten biological replicates were included for each treatment.

### 2.8 Quantification of endogenous phytohormones

To determine the effects of *GhMAPKKK5* silencing on endogenous hormone homeostasis, leaf samples at the eight-leaf stage and young fruiting branch tissues at the squaring stage were collected from CLCrV:00 and CLCrV:*GhMAPKKK5* plants. Approximately 0.5 g of fresh tissue was collected for each biological replicate, and three independent biological replicates were analyzed for each treatment. The contents of brassinosteroids (BRs), cytokinin-related signals, indole-3-acetic acid (IAA), and gibberellic acid (GA_3_) were determined using liquid chromatography-tandem mass spectrometry (LC-MS/MS) according to the method described by Li et al. (2024). Cytokinin-related signals were quantified using 6-benzylaminopurine (6-BA) as the calibration standard and are therefore reported as 6-BA equivalents. Hormone contents were expressed as ng g^-^¹ fresh weight (FW).

### 2.9 RNA extraction and quantitative real-time PCR

Total RNA was extracted using the FastPure Plant Total RNA Isolation Kit (Vazyme, China). First-strand cDNA synthesis was performed using HiScript III RT SuperMix (Vazyme, China). qRT-PCR reactions were conducted on a QuantStudio™ 5 Real-Time PCR System (Applied Biosystems, USA) using 2× Universal SYBR Green Fast qPCR Mix (ABclonal, China). Unless otherwise specified, each qRT-PCR analysis included three independent biological replicates, with three technical replicates performed for each biological replicate using the same cDNA sample. For the VIGS silencing-efficiency assay, equal amounts of tissue from two individual plants were pooled to form one sample, as described in Section 2.7. The mean Ct value of the three technical replicates was used to calculate the relative expression level of each biological replicate. *GhACTIN* was used as the internal reference gene (Zhao et al., 2020), and relative expression levels were calculated using the 2^^−ΔΔCT^ method (Livak and Schmittgen, 2001). Technical replicates were not treated as independent observations in statistical analyses.

### 2.10 Statistical analysis

Statistical analyses were based on independent biological replicates or independently prepared pooled biological samples, as specified for each experiment. Technical qRT-PCR replicates were averaged before statistical analysis and were not treated as independent observations. Unless otherwise specified, each experiment included at least three independent biological replicates. Results are presented as the mean ± standard deviation (SD). Statistical analyses were performed using SPSS v26.0. For comparisons between two groups, homogeneity of variance was assessed using Levene’s test as part of the independent-samples t-test procedure, and the appropriate t-test results were selected according to the outcome of the variance homogeneity test. Differences were considered statistically significant at P < 0.05. Figures were prepared using Origin 2021 and TBtools v2.103.

## 3 Results

### 3.1 Identification, cross-genome synteny, and selective-pressure analyses of *MEKK* genes

To conduct a cross-genome comparison of the *MEKK* subfamily, previously reported cotton MEKK protein sequences were used as queries to identify homologous members in the TM-1, Jin668, Zhongmian113, and Nongdamian8 genomes. A total of 44 MEKK members were identified in each genome (**Supplementary Table 1**), indicating that the number of MEKK family members was generally consistent across the four upland cotton genomes.

To further evaluate the conservation of *MEKK* genes among different upland cotton genomes, whole-genome collinearity and MEKK-specific syntenic relationships were analyzed across the four genomes. Extensive whole-genome synteny was observed, together with numerous MEKK-specific syntenic links between the compared genome pairs (**Figure 1A**). Most corresponding MEKK homologs were located within conserved collinear regions, indicating that the chromosomal positions of *MEKK* genes were generally conserved across the four upland cotton genomes.

**Figure 1:**
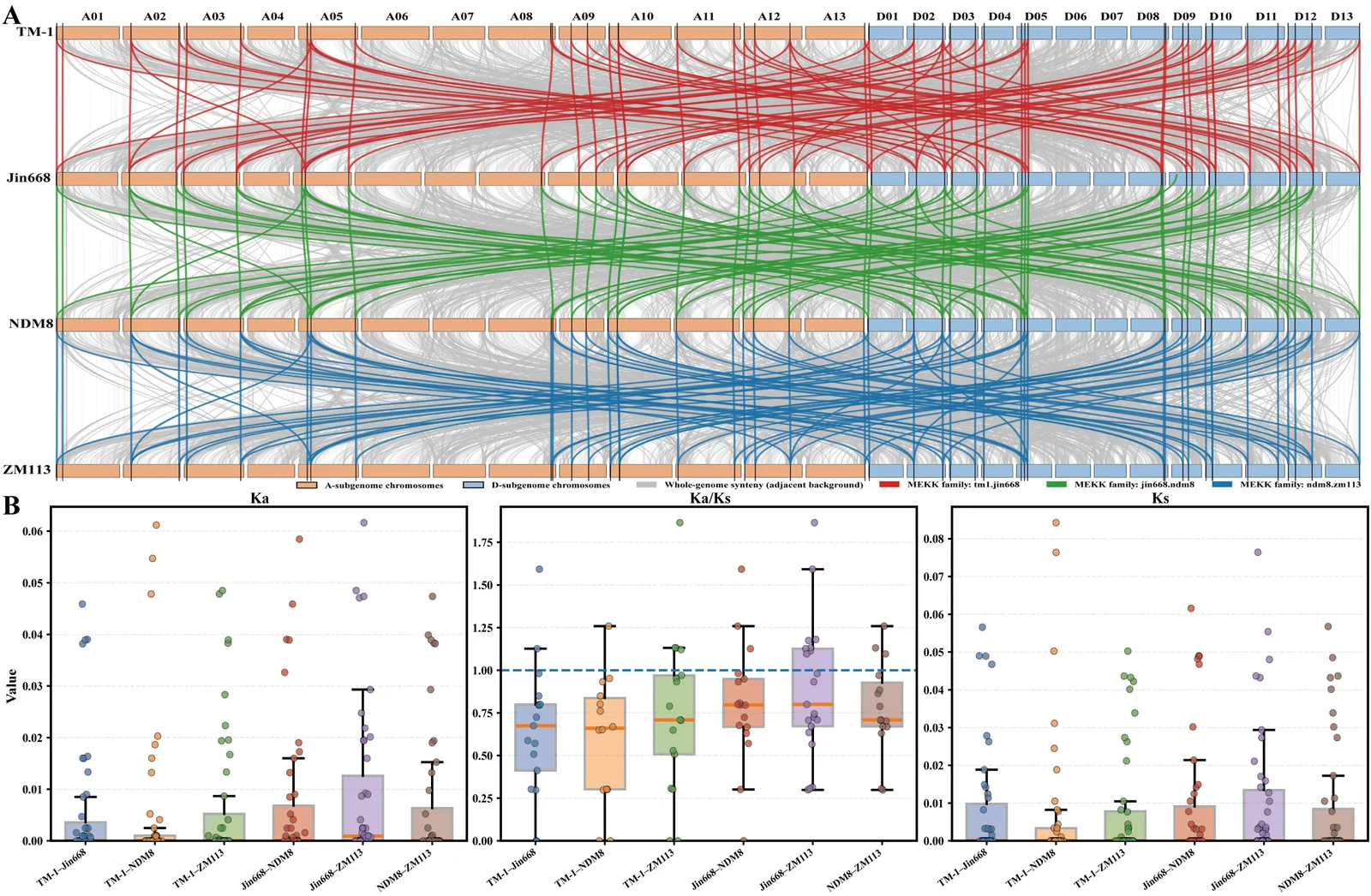
Cross-genome synteny and selective-pressure analyses of MEKK genes in four upland cotton genomes. **(A)** Whole-genome and MEKK-specific syntenic relationships among TM-1, Jin668, Nongdamian8 (NDM8), and Zhongmian113 (ZM113). Orange and blue blocks represent chromosomes of the A and D subgenomes, respectively. Gray curves indicate whole-genome syntenic relationships between adjacent genomes, whereas red, green, and blue curves indicate MEKK-specific syntenic relationships between TM-1 and Jin668, Jin668 and NDM8, and NDM8 and ZM113, respectively. **(B)** Distributions of nonsynonymous substitution rates (Ka), Ka/Ks ratios, and synonymous substitution rates (Ks) for homologous MEKK gene pairs across six pairwise genome comparisons: TM-1-Jin668, TM-1-NDM8, TM-1-ZM113, Jin668-NDM8, Jin668-ZM113, and NDM8-ZM113. Boxplots show the distributions of the calculated values, and individual points represent homologous MEKK gene pairs. The blue dashed line in the Ka/Ks panel indicates Ka/Ks = 1.

Selective-pressure analysis was subsequently performed for homologous *MEKK* gene pairs across all six pairwise comparisons among the four genomes. Most homologous gene pairs exhibited Ka/Ks ratios below 1, and the median Ka/Ks ratio was also below 1 in all six comparisons (**Figure 1B**). These results indicate that the *MEKK* family has predominantly been subjected to purifying selection, suggesting relatively strong evolutionary constraints on the protein-coding sequences of most family members. A small number of homologous *MEKK* gene pairs exhibited Ka/Ks ratios above 1, suggesting that some members may have undergone relatively rapid sequence divergence or experienced altered selective pressures. However, some of these elevated Ka/Ks ratios were accompanied by relatively low Ks values. Therefore, these results should be interpreted cautiously and cannot be considered conclusive evidence of positive selection.

Overall, the number of *MEKK* members was consistent across the four upland cotton genomes, and most *MEKK* genes showed strong conservation of genomic position, together with predominantly purifying selection acting on their coding sequences. These results indicate that the *MEKK* subfamily is generally highly conserved across the four upland cotton genomes, although a subset of members may have undergone a certain degree of sequence divergence.

### 3.2 Gene structure and conserved motif analysis of *MEKK* subfamily members in upland cotton

To further investigate differences in gene structure among the identified MEKK homologs, the exon-intron organization and conserved protein motifs of corresponding MEKK genes across the four genomes were comparatively analyzed. Most corresponding *MEKK* homologs exhibited broadly similar exon-intron organization across the four genomes, whereas several homologous groups showed differences in exon number or intron length (**Supplementary Figure S1**). These results indicate that, although the *MEKK* family is generally conserved at the cross-genome level, local variation in gene structure is still present among some members.

Conserved-domain analysis further showed that all identified MEKK proteins contained the characteristic S_TKc domain (**Supplementary Figure S2A**), indicating strong conservation of the core kinase domain within the *MEKK* family. In contrast, motif analysis revealed variation in the composition and arrangement of several motifs among corresponding homologous proteins from different genomes (**Supplementary Figure S2B**). Among the ten motifs identified by MEME, motifs 4, 5, 8, 9, and 10 showed the greatest variation. Motifs 4 and 5 were mainly characterized by motif loss in one or more corresponding homologs, whereas motifs 8, 9, and 10 exhibited both motif loss and positional variation (**Figure 2**).

**Figure 2:**
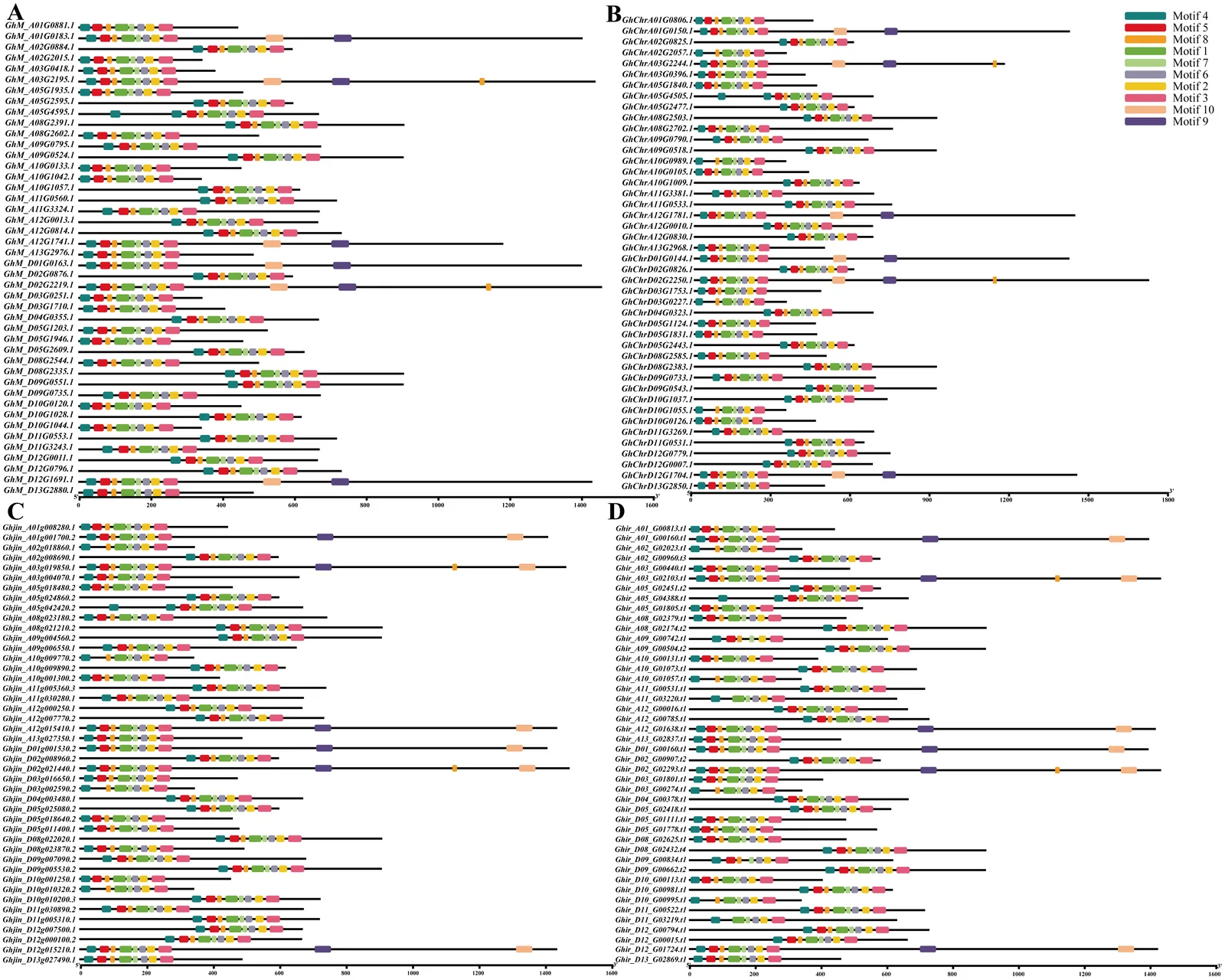
Structural features of MEKK subfamily members in four upland cotton genomes. **(A–D)** Distribution of conserved motifs in GhMEKK proteins from four upland cotton genomes, including Nongdamian8 **(A)**, TM-1 **(B)**, Jin668 **(C)**, and Zhongmian113 **(D)**.

Based on the cross-genome comparison of motif composition and arrangement, the 44 *MEKK* homologous groups were further classified into motif-conserved and motif-variable groups. Among them, 26 homologous groups showed relatively consistent motif composition and arrangement across the four genomes and were therefore classified as motif-conserved groups. The remaining 18 homologous groups exhibited loss or positional variation of one or more motifs in at least one genome and were classified as motif-variable groups. Overall, the *MEKK* family showed a high degree of conservation at the levels of genomic position, coding sequence, and core kinase domain. Nevertheless, some homologous members exhibited local divergence in exon-intron organization and motif composition and arrangement. These findings indicate that, despite the overall strong evolutionary constraints acting on the *MEKK* family, local differences in exon-intron organization and motif architecture are present among some corresponding homologous members.

### 3.3 Phylogenetic relationships and promoter *cis*-regulatory features of *MEKK* genes

To further investigate the evolutionary relationships among the identified MEKK members, a phylogenetic tree was constructed using MEKK proteins from the four upland cotton genomes. The results showed that the MEKK proteins could be divided into four major clades (**Figure 3A**). Corresponding homologs from different genomes generally clustered within the same or closely related subclades, consistent with the high degree of cross-genome conservation revealed by the synteny and selective-pressure analyses described above.

**Figure 3:**
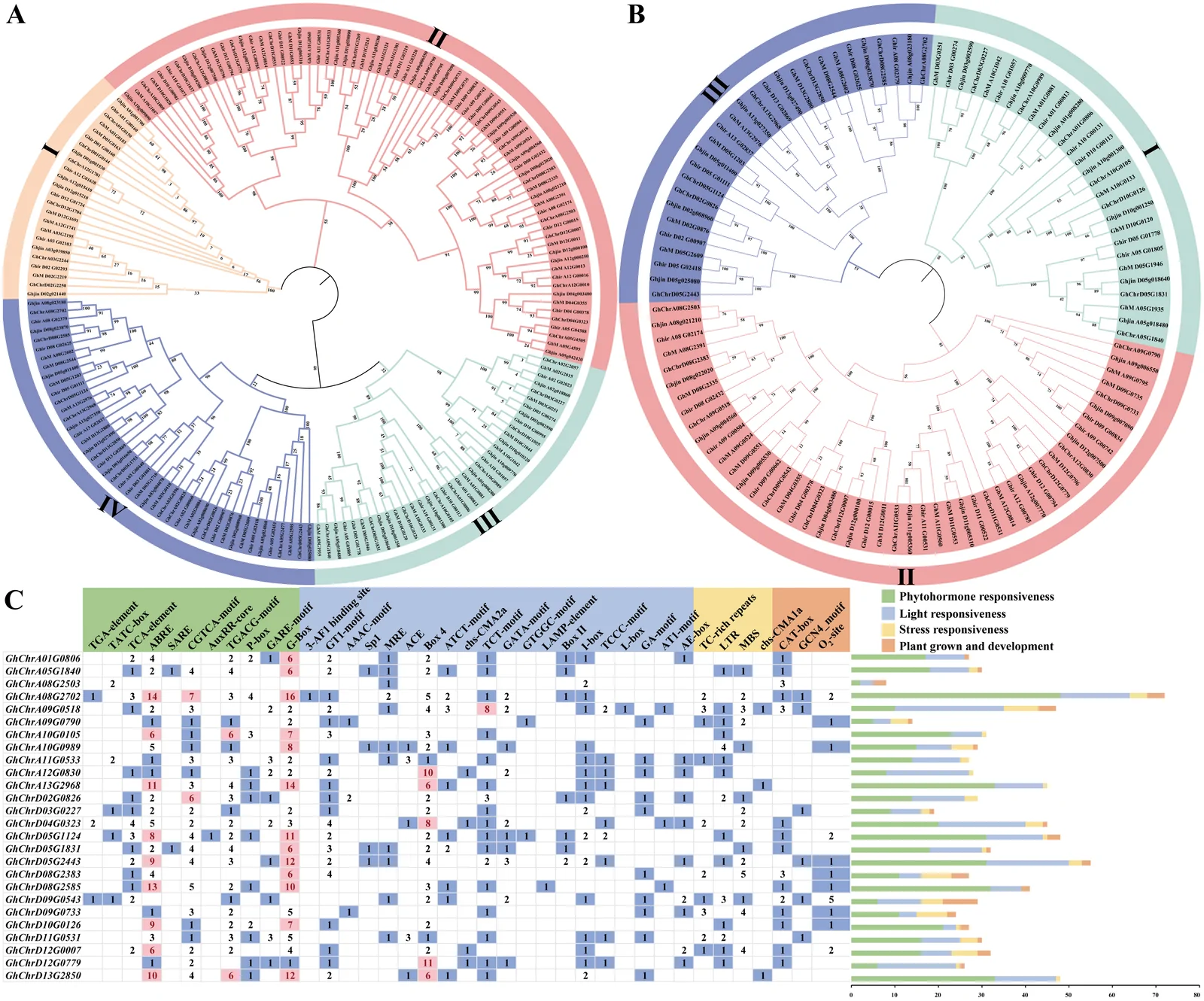
Phylogenetic relationships and promoter *cis*-regulatory features of *MEKK* genes in upland cotton. **(A)** Phylogenetic relationships among MEKK proteins identified from the TM-1, Jin668, Zhongmian113, and Nongdamian8 genomes. Different background colors indicate the four major phylogenetic clades. **(B)** Phylogenetic relationships among MEKK proteins corresponding to the 26 motif-conserved homologous groups. Different background colors indicate the three major phylogenetic clades. **(C)** Distribution of *cis*-acting regulatory elements in the 2-kb upstream promoter regions of the corresponding TM-1 genes from the 26 motif-conserved *MEKK* homologous groups. Numbers within the heatmap indicate the copy number of each *cis*-element. The identified elements were classified into four functional categories: phytohormone responsiveness, light responsiveness, stress responsiveness, and plant growth and development. Stacked bars on the right indicate the total numbers of *cis*-elements in each functional category for individual genes.

The relationship between phylogenetic classification and motif conservation was further examined. Members corresponding to the 26 motif-conserved MEKK homologous groups were mainly distributed among three major phylogenetic clades (**Figure 3B**). Most motif-conserved groups were distributed in clades II, III, and IV, whereas clade I was mainly composed of motif-variable members. In addition, several motif-variable homologous groups were also distributed within clades II-IV, indicating that motif conservation was not completely consistent with phylogenetic classification.

To further characterize the potential transcriptional regulatory features of the motif-conserved *MEKK* members, the 2-kb upstream promoter regions of the corresponding TM-1 genes from these 26 homologous groups were analyzed for *cis*-acting regulatory elements. A wide range of *cis*-elements associated with light responsiveness, phytohormone responses, stress responses, and growth and development were identified (**Figure 3C**). Light-responsive elements were detected in all 26 promoters, whereas phytohormone-responsive elements represented the most abundant functional category. In total, 149 G-box elements and 122 ABRE elements were identified across the analyzed promoter regions. The number and composition of *cis*-acting elements varied among individual genes. *GhChrA08G2702*, *GhChrA13G2968*, and *GhChrD13G2850* contained relatively large numbers of predicted *cis*-elements, whereas *GhChrA08G2503*, *GhChrA09G0790*, and *GhChrD12G0779* contained comparatively fewer *cis*-elements. These results indicate that the motif-conserved MEKK members differ to some extent in their predicted promoter regulatory features. However, the presence or abundance of predicted *cis*-elements alone does not provide direct evidence of their functional activity.

Taken together, the phylogenetic and promoter analyses provide complementary information on the evolutionary relationships and potential transcriptional regulatory characteristics of MEKK members and provide a basis for subsequent expression-based candidate-gene screening.

### 3.4 Identification of key candidate genes associated with fruiting branch angle in upland cotton

To further screen candidate genes associated with fruiting branch angle (FBA) from the 26 motif-conserved *MEKK* homologous groups, transcriptome data from the fifth fruiting branches of the large-FBA accession Shaan689 and the small-FBA accession Jimian8 were first analyzed. Expression differences of the corresponding *MEKK* genes between the two accessions were compared. The expression ratio of Jimian8 to Shaan689 was used as the initial screening criterion. The 26 MEKK genes showed varying degrees of expression differences between the two accessions (**Figure 4A**). Using an expression ratio of >1.5 or <0.5 as the threshold, eight genes with relatively large expression differences were selected for further analysis.

**Figure 4:**
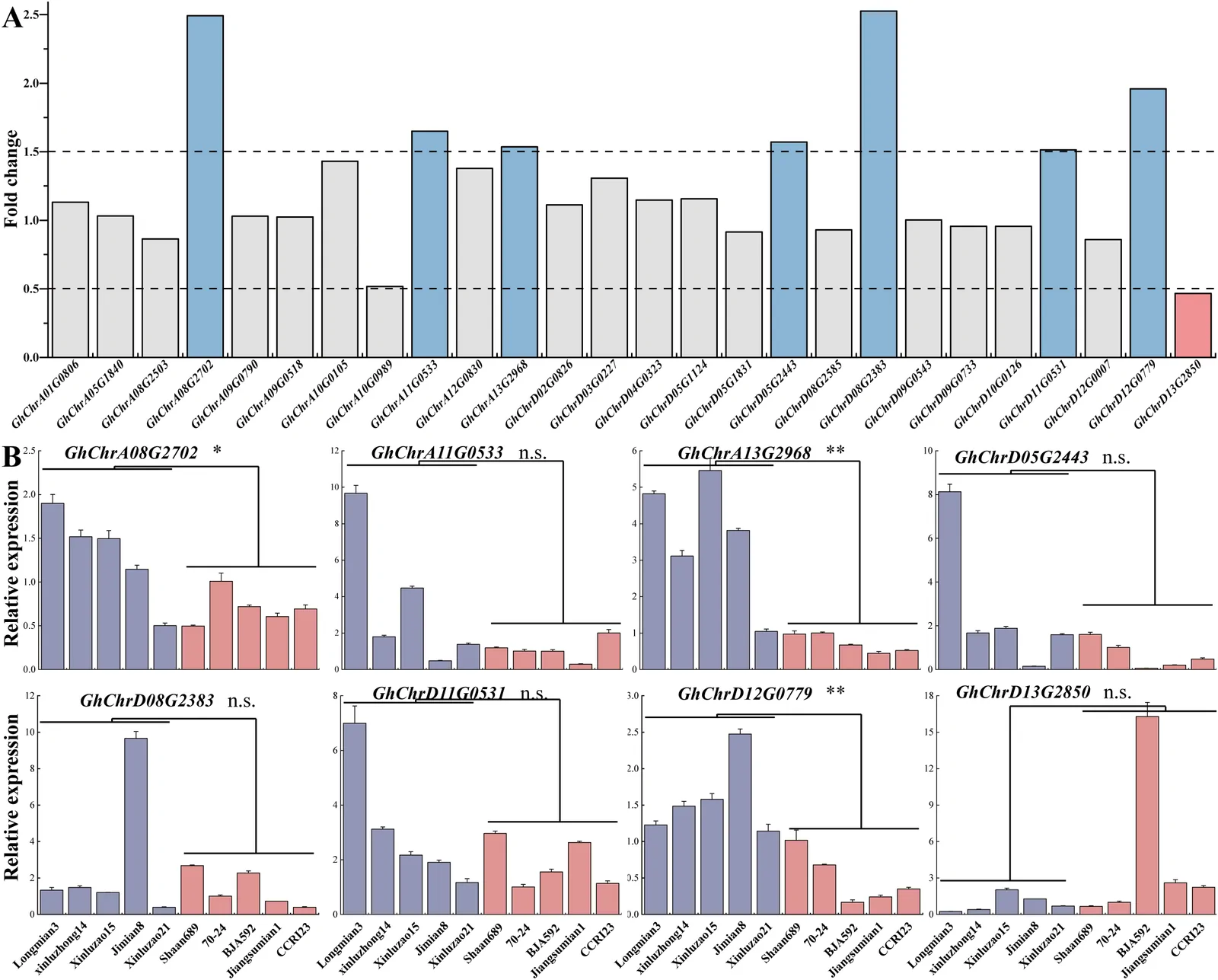
Transcriptome-based screening and qRT-PCR validation of candidate *MEKK* genes associated with fruiting branch angle in upland cotton. **(A)** Expression differences of 26 motif-conserved *MEKK* genes between two cotton accessions based on transcriptome data. Dashed lines indicate the screening thresholds of 1.5 and 0.5. Blue bars indicate genes with expression ratios >1.5, the red bar indicates a gene with an expression ratio <0.5, and gray bars indicate genes with expression ratios between 0.5 and 1.5. **(B)** qRT-PCR validation of the eight selected genes in five relatively small-FBA accessions and five relatively large-FBA accessions. Blue-purple bars represent the small-FBA accessions, whereas red bars represent the large-FBA accessions. Differences between the two FBA groups were evaluated using an independent-samples *t*-test after assessment of variance homogeneity using Levene’s test. *P < 0.05; **P < 0.01; *n.s*., not significant. Error bars represent the standard deviation of three independent biological replicates (n = 3).

To validate the transcriptome-based screening results, the expression levels of these eight genes were further examined by qRT-PCR. Five relatively large-FBA accessions and five relatively small-FBA accessions were included as contrasting phenotypic groups (**Figure 4B**). Among the eight genes, *GhChrA08G2702*, *GhChrA13G2968*, and *GhChrD12G0779* showed significant expression differences between the two FBA groups. No significant differences were detected for the other five genes. *GhChrA08G2702* generally showed higher expression in the small-FBA accessions than in the large-FBA accessions. Similarly, *GhChrA13G2968* and *GhChrD12G0779* were expressed at higher levels in the small-FBA group, with differences reaching *P* < 0.01.

The transcriptome comparison between Shaan689 and Jimian8 was then considered together with the qRT-PCR results obtained from the contrasting FBA accessions. *GhChrD12G0779* showed a consistent expression pattern associated with FBA variation in both analyses. Its expression difference remained significant after validation across a broader set of cotton accessions. Therefore, based on the combined results of the two-step expression screening, *GhChrD12G0779 (GhMAPKKK5)* was prioritized for subsequent functional validation.

### 3.5 Silencing of *GhMAPKKK5* significantly increases fruiting branch angle in upland cotton

To determine whether *GhMAPKKK5* is involved in FBA regulation, a VIGS assay was performed in the compact cotton accession Jimian8. Approximately 30 days after infiltration, plants carrying the positive control construct CLCrVA:*GhCHLI* exhibited a typical photobleaching phenotype, indicating that the VIGS system was functioning effectively (**Supplementary Figure S3A**). Quantitative RT-PCR analysis further confirmed efficient silencing of *GhMAPKKK5*, with most CLCrV:*GhMAPKKK5* plants exhibiting transcript levels reduced to less than 50% of those observed in CLCrV:00 plants (**Supplementary Figure S3B**).

To evaluate the phenotypic consequences of *GhMAPKKK5* silencing, fruiting branch angles of lower and upper fruiting branches were measured at both the squaring-to-flowering stage and the boll-setting stage (**Figures 5A, B**). At the squaring-to-flowering stage, the average angle of lower fruiting branches increased from 67.89° in CLCrV:00 plants to 71.09° in CLCrV: *GhMAPKKK5* plants, whereas the angle of upper fruiting branches increased from 70.32° to 73.88° (**Figure 5C**). Similar trends were observed at the boll-setting stage, where the lower and upper fruiting branch angles increased by 3.65° and 3.99°, respectively, following *GhMAPKKK5* silencing (**Figure 5D**).

**Figure 5:**
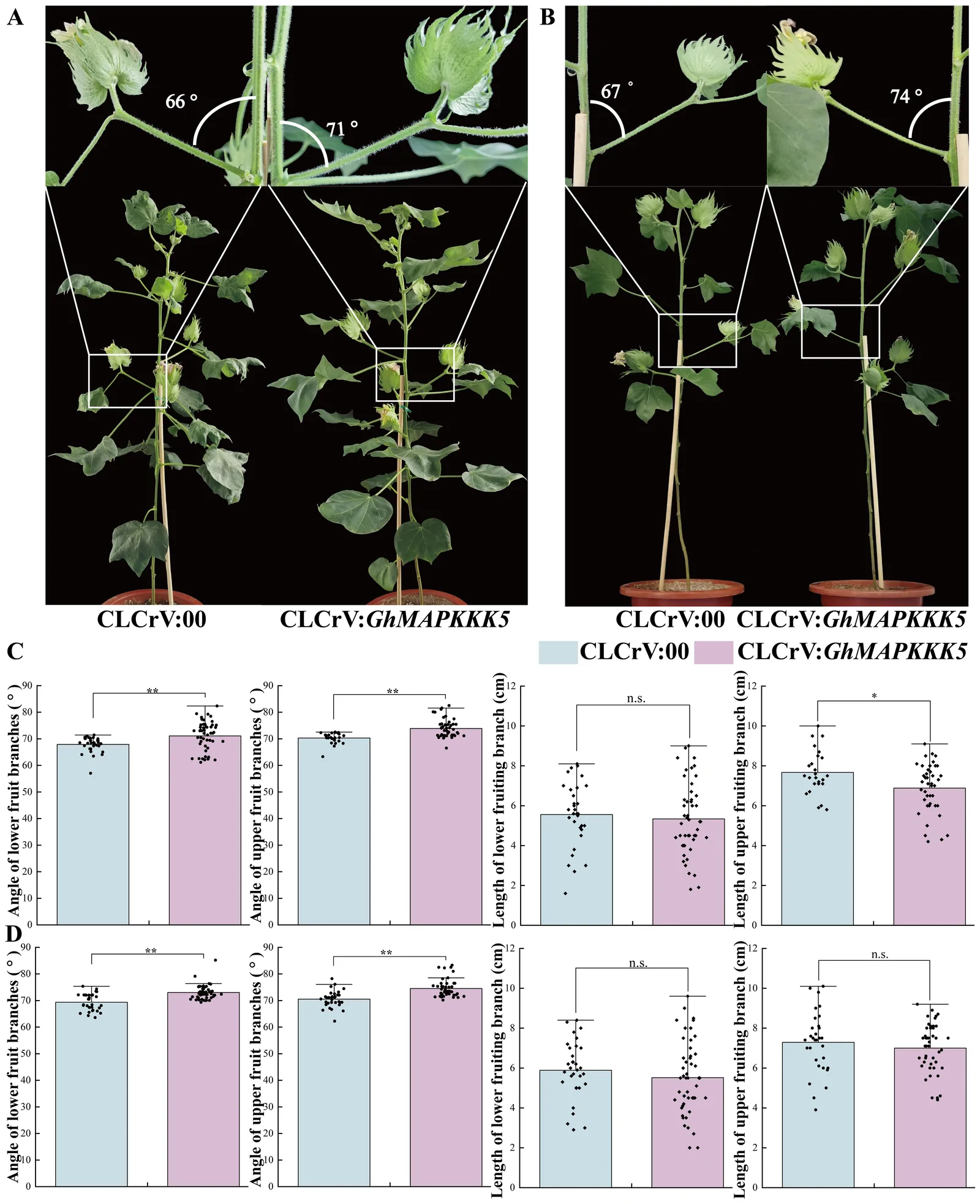
Effects of *GhMAPKKK5* on fruiting branch angle and fruiting branch length. **(A, B)** Representative fruiting branch angle phenotypes of CLCrV:00 and CLCrV:*GhMAPKKK5* plants at the squaring-to-flowering stage **(A)** and boll-setting stage **(B–D)** Statistical analysis of fruiting branch angle and fruiting branch length at the squaring-to-flowering stage **(C)** and boll-setting stage **(D)**. Significant differences between CLCrV:00 and CLCrV:*GhMAPKKK5* plants were determined using Student’s *t*-test (*P < 0.05, **P < 0.01); n.s., not significant. Sample size, *n* ≥ 10.

In contrast, fruiting branch length was only marginally affected by *GhMAPKKK5* suppression. No significant differences were detected in lower fruiting branch length at the squaring-to-flowering stage, and branch lengths of both lower and upper fruiting branches remained comparable between silenced and control plants at the boll-setting stage (**Figures 5C, D**).

Together, these data show that transient suppression of *GhMAPKKK5* increased FBA across two developmental stages without substantially affecting branch elongation. The findings provide preliminary functional evidence that *GhMAPKKK5* negatively influences FBA in the Jimian8 genetic background.

### 3.6 *GhMAPKKK5* silencing is associated with altered endogenous hormone contents in cotton plants

Phytohormone homeostasis strongly influences plant architecture. We therefore tested whether the enlarged FBA phenotype in *GhMAPKKK5*-silenced plants was associated with altered hormone levels. Major phytohormones were quantified in leaves at the eight-leaf stage and in young fruiting branches at the squaring stage.

In leaves, silencing of *GhMAPKKK5* significantly increased the BR content from 7.54 ng g^-^¹ FW in CLCrV:00 plants to 11.47 ng g^-^¹ FW in CLCrV:*GhMAPKKK5* plants, representing an increase of 52.1%. In contrast, the cytokinin-equivalent content, expressed as 6-BA equivalents, decreased from 3.51 to 2.01 ng g^-^¹ FW, corresponding to a reduction of 42.7%, while the IAA content decreased from 2.14 to 0.99 ng g^-^¹ FW, representing a reduction of 53.7% (**Figure 6A**). No significant difference in GA_3_ content was detected between the two treatments. In young fruiting branches, the BR content increased from 2.21 ng g^-^¹ FW in CLCrV:00 plants to 2.98 ng g^-^¹ FW in CLCrV:*GhMAPKKK5* plants, representing an increase of 34.8%. Conversely, the CK-equivalent content decreased from 0.25 to 0.16 ng g^-^¹ FW, corresponding to a reduction of 36.0% (**Figure 6B**). The contents of GA_3_ and IAA in young fruiting branches remained largely unchanged.

**Figure 6:**
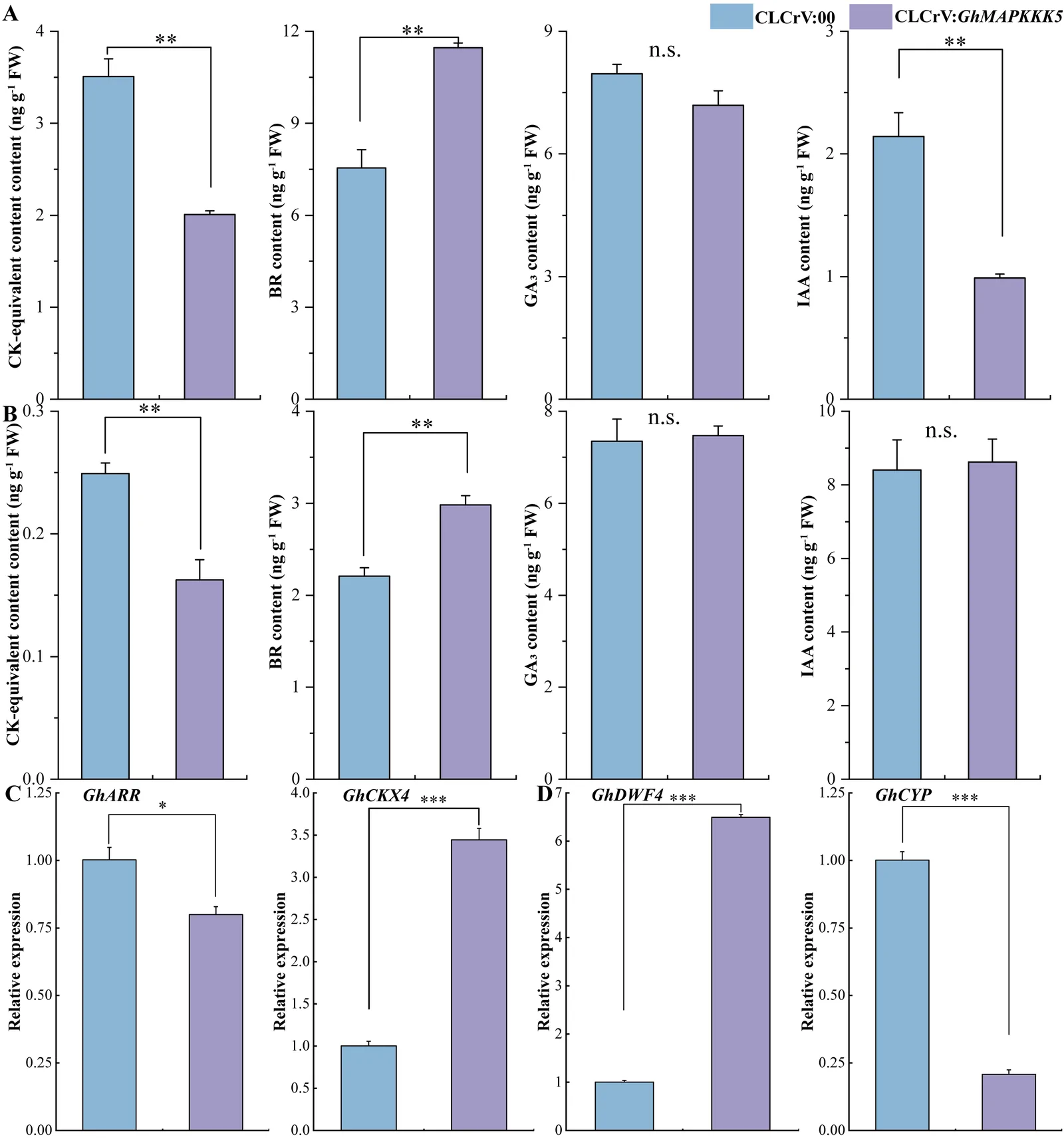
Endogenous hormone contents and related gene expression in *GhMAPKKK5* silenced plants.**(A, B)** Contents of BRs, CK equivalents expressed as 6-BA equivalents, IAA, and GA_3_ in leaves **(A)** and young fruiting branches **(B)** of CLCrV:00 and CLCrV:*GhMAPKKK5* plants. **(C, D)** Relative expression levels of genes associated with cytokinin signaling and metabolism **(C)** and BR biosynthesis and metabolism **(D)** in CLCrV:00 and CLCrV: *GhMAPKKK5* plants. Differences between CLCrV:00 and CLCrV:GhMAPKKK5 plants were evaluated using an independent-samples t-test after assessment of variance homogeneity using Levene’s test. Error bars represent the standard deviation of three independent biological replicates (n = 3).*P < 0.05, **P < 0.01, ***P < 0.001; n.s., not significant.

To further determine whether these hormonal changes were associated with altered expression of hormone-related genes, several key genes involved in BR and cytokinin biosynthesis or metabolism were examined by qRT-PCR. In the cytokinin pathway, the expression of *GhARR*, a cytokinin-responsive gene, was significantly decreased, whereas *GhCKX4*, which encodes a cytokinin oxidase/dehydrogenase involved in cytokinin degradation, was significantly upregulated in *GhMAPKKK5*-silenced plants (**Figure 6C**). In the BR pathway, the BR biosynthetic gene *GhDWF4* showed significantly increased expression, whereas the BR catabolism-related gene *GhCYP* was significantly downregulated (**Figure 6D**).

Thus, *GhMAPKKK5* silencing was associated with increased brassinosteroid content, reduced cytokinin-equivalent content, and altered expression of hormone-related genes. These correlations link *GhMAPKKK5* activity to brassinosteroid- and cytokinin-related processes but do not establish direct regulation of hormone homeostasis or signaling. The changes may instead be secondary responses to altered fruiting branch development.

### 3.7 *GhMAPKKK5* silencing is associated with altered expression of branch angle-related genes

Because *GhMAPKKK5* silencing significantly increased fruiting branch angle and altered endogenous hormone levels, we further investigated whether this phenotype was accompanied by changes in the expression of genes previously implicated in branch angle or plant architecture regulation. A set of candidate genes associated with branch orientation, gravitropic responses, and plant architecture was selected for expression analysis, including *GhSAUR* (Shao et al., 2022), *GhLAZY1* (Shao et al., 2022), *GhTAC1* (Ku et al., 2011; Dardick et al., 2013; Xu et al., 2017; Li et al., 2022; Yin et al., 2025), *GhDREB1B* (Ji et al., 2021), *GhDCA1* (Wang et al., 2025c), and *GhSGR5* (Morita et al., 2006). These genes were either identified as candidate regulators of FBA in cotton or were homologs of genes known to regulate branch or tiller angle in other plant species.

Compared with CLCrV:00 control plants, the transcript levels of *GhSAUR*, *GhLAZY1*, and *GhTAC1* were significantly increased in *GhMAPKKK5*-silenced plants (**Figure 7A**). In contrast, the expression levels of *GhDREB1B*, *GhDCA1*, and *GhSGR5* were significantly reduced following *GhMAPKKK5* suppression (**Figure 7B**). Previous studies have shown that LAZY1, TAC1, and SAUR family genes are involved in organ orientation, gravitropic responses, and branch angle formation. In the present study, the enlarged FBA phenotype following *GhMAPKKK5* silencing was accompanied by increased transcript levels of *GhSAUR*, *GhLAZY1*, and *GhTAC1* and reduced transcript levels of *GhDREB1B*, *GhDCA1*, and *GhSGR5*. These results indicate that *GhMAPKKK5* silencing is associated with transcriptional changes in several genes related to gravitropism and plant architecture. However, these expression changes do not demonstrate direct regulation by *GhMAPKKK5*.

**Figure 7:**
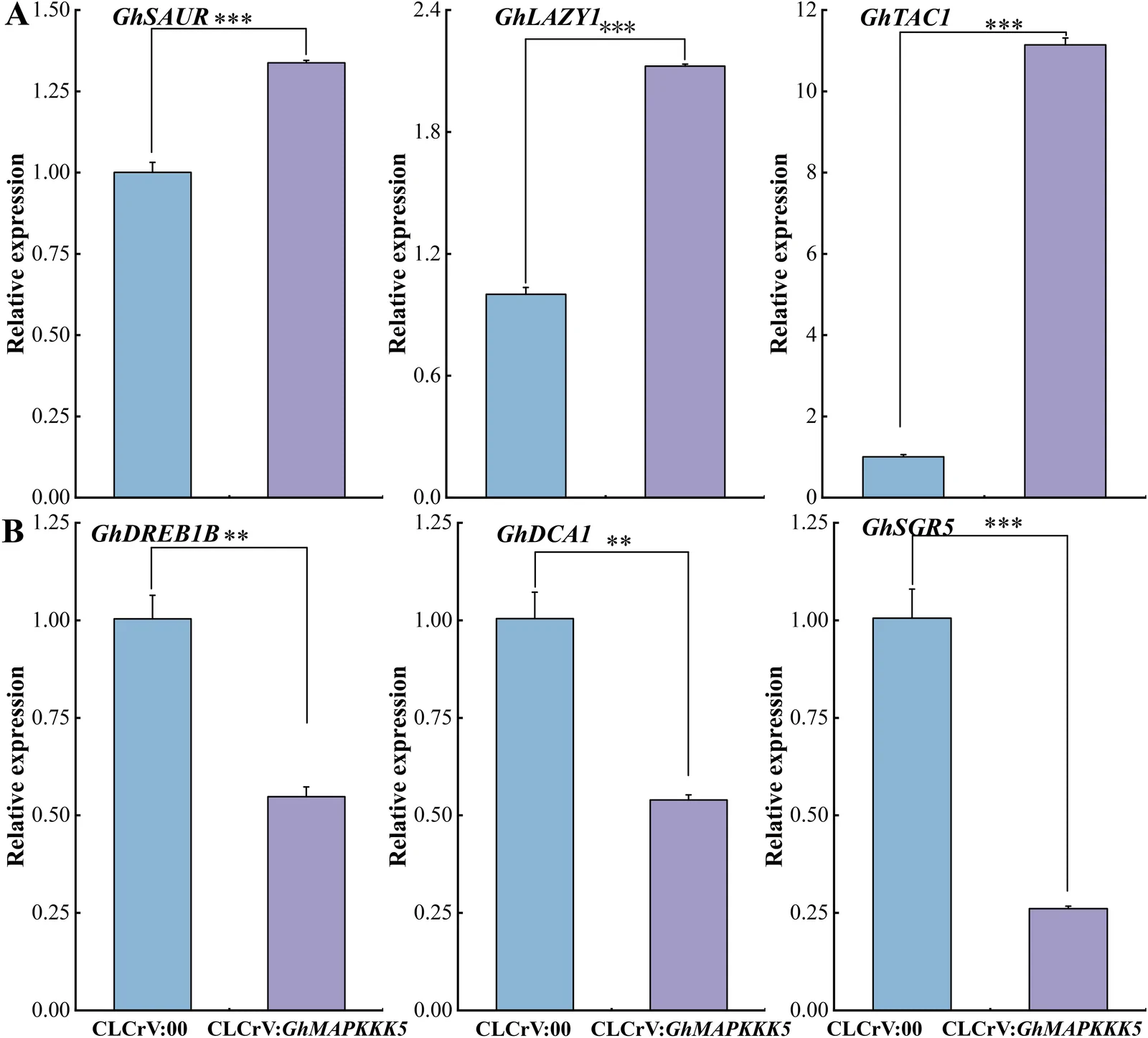
Expression analysis of candidate branch angle-associated genes in CLCrV:*GhMAPKKK5* plants. **(A, B)** Relative expression levels of candidate branch angle-associated genes that were upregulated **(A)** or downregulated **(B)** in CLCrV: *GhMAPKKK5* plants compared with CLCrV:00 controls. Differences between the two groups were evaluated using an independent-samples t-test after assessment of variance homogeneity using Levene’s test. *P < 0.05; **P < 0.01; ***P < 0.001. Error bars represent SD (*n* = 3).

Overall, *GhMAPKKK5* silencing altered the expression of several branch angle-related genes. Together with the hormone data, these findings show that the enlarged FBA phenotype coincided with changes in hormone status and transcriptional responses associated with gravitropism and plant architecture. The qRT-PCR data cannot distinguish direct regulation from indirect or secondary responses. Molecular and genetic analyses are needed to determine whether *GhMAPKKK5* and these genes act in a common pathway.

## 4 Discussion

With the development of high-quality genome assemblies and pan-genome resources, multi-genome comparisons have become useful for identifying intraspecific variation that may be overlooked when relying on a single reference genome (Shi et al., 2023). Pan-genome analyses of the *TIFY*, *NAC*, *GT64*, and *OXS3* families have revealed variation in gene copy number, gene structure, conserved motifs, protein features, and expression patterns, thereby providing insights into gene family evolution and functional divergence (Liu et al., 2024; Lu et al., 2024; Zhao et al., 2024; Wang et al., 2025b). In the present study, *MEKK* members were compared across the Jin668, Zhongmian113, TM-1, and Nongdamian8 genomes. All identified proteins contained the conserved S_TKc domain, whereas variation was observed in gene structure and motif composition, particularly in motifs 4, 5, 8, 9, and 10. Cross-genome synteny analysis further revealed extensive conservation of MEKK loci among the four upland cotton genomes. In addition, most homologous *MEKK* gene pairs exhibited Ka/Ks ratios below 1, indicating that the family has predominantly evolved under purifying selection. Together, these results suggest that *MEKK* genes are generally subject to strong evolutionary constraints, although local variation in exon-intron organization and motif architecture has occurred among a subset of homologous members. Based on motif composition and arrangement, 26 homologous groups were classified as motif-conserved and 18 as motif-variable. Phylogenetic and promoter analyses further indicated both evolutionary conservation and potential functional divergence among *MEKK* members. The abundance of light- and phytohormone-responsive *cis*-elements is consistent with the established roles of light, gravity, auxin, BRs, and CKs in regulating plant architecture and organ angle (Bai et al., 2007; Luo et al., 2016; Tian et al., 2019; He et al., 2021; Yuan et al., 2023; Chen et al., 2024; Ali et al., 2025; Zhang et al., 2025). Compared with previous cotton MAPKKK studies based on a more limited reference-genome framework, the four-genome comparison reduced reliance on a single reference genome and provided an additional basis for assessing which MEKK features were consistently retained across the four independently assembled genomes. By integrating cross-genome conservation, phylogenetic relationships, and expression information, this multi-genome comparative framework linked *MEKK* family comparison with phenotype-associated candidate prioritization. However, the present homolog-based comparison was not designed as a systematic genome-wide analysis of presence/absence variation (PAV) or structural variation (SV). Because the four reference genomes were independently assembled and annotated, apparent gene absence or structural differences may be influenced by differences in genome assembly or gene annotation. Rigorous characterization of PAV and SV will require standardized genome-to-genome comparisons, harmonized annotations, and dedicated variant-detection pipelines.

In cotton, fruiting branch length, the height of the first fruiting branch node, and fruiting branch angle (FBA) are important architecture-related traits that affect canopy light interception, light-use efficiency, and dry matter accumulation (Wu, 2012). Although QTL mapping, GWAS, and transcriptome analyses have identified several loci and candidate genes associated with FBA, most remain functionally unverified (Shao et al., 2022; Tan et al., 2025). In the present study, *GhMAPKKK5* was prioritized from the motif-conserved MEKK group by integrating cross-genome classification, phylogenetic relationships, and differential expression between accessions with contrasting FBA phenotypes. VIGS-mediated silencing of *GhMAPKKK5* in Jimian8 increased FBA at both the squaring-flowering and boll-setting stages, whereas fruiting branch length was only slightly affected. These findings provide preliminary functional support for a negative association between *GhMAPKKK5* activity and FBA. MAPK cascades have also been implicated in the regulation of organ development and plant architecture-related traits in rice, further supporting a potential connection between MAPK signaling and cotton branch architecture (Duan et al., 2014; Liu et al., 2015, Liu et al., 2021; Yan et al., 2025). Recent advances in the genetics of cotton plant architecture provide an important context for interpreting these results. CRISPR/Cas9-mediated disruption of the A11/D11 *GhTAC1* homeologs reduced sympodial branch and petiole angles, providing direct genetic evidence that the conserved TAC1 pathway contributes to cotton shoot orientation (Kangben et al., 2024). More recently, map-based cloning of qFBA-A11 identified *GhFBA1_At* as a positive regulator of FBA. The GATA5-*GhFBA1_At*-KNAT7 module was shown to regulate cell expansion and cell-wall thickness, and CRISPR/Cas9-mediated editing of *GhFBA1_At* produced a more compact architecture and improved yield under high-density planting (Xu et al., 2026). In parallel, GWAS and transcriptomic analyses have implicated gravity-, light-, and auxin-responsive processes, including a SAUR46 homolog, in natural variation in cotton FBA (Shao et al., 2022). LAZY proteins are important components of gravity signaling and auxin redistribution in other plant species, but direct genetic evidence linking *GhLAZY1* to cotton FBA remains limited. Hormone-dependent regulation of cotton architecture has also been reported for *GhAPC8*, whose silencing altered BR, auxin, and GA contents and reduced leaf blade angle (Jin et al., 2022). Against this background, the increased FBA observed after *GhMAPKKK5* silencing suggests that *GhMAPKKK5* may represent a distinct negative input into branch-angle regulation, contrasting with the positive contributions of *GhTAC1* and *GhFBA1_At* to branch inclination. However, the accompanying changes in *GhLAZY1*, *GhTAC1*, *GhSAUR*, and hormone-related traits remain correlative, and the present results do not establish whether *GhMAPKKK5* functions within, upstream of, or independently from these previously identified architecture-related pathways.

The functional evidence presented here is based on VIGS-mediated transient silencing, which produces temporary and potentially incomplete suppression rather than a stable, heritable knockout. The consistent increase in FBA across two developmental stages and multiple branch positions nevertheless supports a putative negative role for *GhMAPKKK5*. Validation was limited to the small-angle accession Jimian8, and the magnitude or direction of the response may differ among genetic backgrounds. Stable overexpression, CRISPR/Cas-mediated knockout, and complementation studies in multiple backgrounds are therefore needed to confirm gene function. Because FBA is environmentally responsive, results obtained under controlled conditions do not demonstrate environmental stability. Multi-environment trials should assess the robustness of the phenotype across locations, seasons, and cultivation systems. Yield components, fiber quality, flowering time, reproductive development, and overall fitness must also be evaluated before *GhMAPKKK5* can be considered for breeding applications.

Previous studies have shown that MAPK cascades can interact with BR signaling in rice. *OsMAPK6* influences BR homeostasis and BR sensitivity, whereas *OsMAPKKK5* affects BR signaling by phosphorylating OsBSK1–1 and altering OsBZR1 nuclear localization (Liu et al., 2015; Wang et al., 2025a; Yan et al., 2025). In cotton, *GhMAPKKK5* silencing was associated with increased BR content and altered expression of BR-related genes. The reduction in cytokinin-equivalent content, expressed as 6-BA equivalents, together with the downregulation of *GhARR* and upregulation of *GhCKX4*, further suggests that cytokinin-related signaling and metabolism may also be altered. Silencing of *GhMAPKKK5* was also accompanied by increased transcript levels of *GhSAUR*, *GhLAZY1*, and *GhTAC1* and reduced transcript levels of *GhDREB1B*, *GhDCA1*, and *GhSGR5*. The altered transcript levels of these genes represent associated transcriptional responses rather than evidence that they are directly regulated by *GhMAPKKK5*. Because *GhMAPKKK5* is a protein kinase rather than a known DNA-binding transcription factor, direct promoter binding is not assumed. The observed expression changes may instead result from downstream kinase signaling, altered hormone status, or secondary responses associated with fruiting branch development.

MAPKKKs generally act upstream of MAPKK-MAPK modules, but the downstream MAPKK and MAPK components of *GhMAPKKK5* were not identified or validated here. The study also did not test direct protein interactions, substrate phosphorylation, promoter regulation by downstream transcription factors, or genetic epistasis. The current evidence therefore neither defines a specific *GhMAPKKK5*-MAPKK-MAPK cascade nor distinguishes direct from secondary responses. Protein-interaction assays, *in vitro* kinase analyses, phosphoproteomic profiling, hormone-response experiments, and promoter regulation assays will be required to resolve the pathway. Stable genetic materials will further clarify how this pathway influences cotton FBA.

## 5 Conclusions

Comparative analysis of the cotton MEKK family identified 26 motif-conserved homologous groups across four upland cotton genomes. Integrating cross-genome classification with expression analysis prioritized *GhMAPKKK5* as a candidate associated with fruiting branch angle (FBA). Transient silencing of *GhMAPKKK5* increased FBA and coincided with changes in brassinosteroid and cytokinin-equivalent contents and in the expression of hormone- and branch angle-related genes. These findings provide preliminary evidence that *GhMAPKKK5* negatively influences cotton FBA, but they do not establish its molecular mechanism. Stable genetic validation and field evaluation are needed to determine its effects on architecture, yield, fiber quality, and agronomic performance. *GhMAPKKK5* is therefore a candidate for further mechanistic and breeding-oriented evaluation.

## Data Availability

The original contributions presented in the study are included in the article/**Supplementary Material**. Further inquiries can be directed to the corresponding authors.

## Supplementary material

Supplementary Figure 1 Gene structures of *GhMEKK* genes in four upland cotton genomes.

Supplementary Figure 2 Conserved domain and motif features of GhMEKK proteins.

Supplementary Figure 3 CLCrV:*GhCHLI* phenotype and relative expression levels in CLCrV:*GhMAPKKK5* plants.

Supplementary Figure 4 Standard curves of 6-BA, BRs, GA3, and IAA.

Supplementary Table 1 Gene IDs of MEKK subfamily members identified in the Jin668, TM-1, Zhongmian113, and Nongdamian8 genomes.

Supplementary Table 2 Phenotypic values of cotton accessions with relatively large and relatively small FBA.
